# Dual-mechanism anti-CD73 antibodies CR201 and CR202 targeting distinct domains for cancer immunotherapy

**DOI:** 10.3389/fimmu.2026.1861537

**Published:** 2026-06-10

**Authors:** Miao Zhang, Haibin Yuan, Xindi Pan, Xian Li, Zichen Wang, Shuping Zhang, Zhigang Gu, Biao Hu, Xuedong Qu, Qian Wang, Xiangguo Gu, Bo Wang, Yu Cao, Guilin Mu, Guangbo Kang, Ario de Marco, Xiangshan Zhou, He Huang

**Affiliations:** 1 School of Synthetic Biology and Biomanufacturing, State Key Laboratory of Synthetic Biology, Tianjin Key Laboratory of Biological and Pharmaceutical Engineering, Tianjin University, Tianjin, China; 2 China Resources Biopharmaceutical Company Limited, Shenzhen, China; 3 Laboratory for Environmental and Life Sciences, University of Nova Gorica, Nova Gorica, Slovenia

**Keywords:** cancer immunotherapy, CD73, enzymatic inhibition, internalization, monoclonal antibody, tumor microenvironment

## Abstract

**Background:**

CD73 is a key enzyme in the adenosine-mediated immunosuppressive pathway and represents an attractive target for cancer immunotherapy. Here, we aimed to develop novel anti-CD73 antibodies with dual mechanisms of action by simultaneously inhibiting enzymatic activity and promoting receptor internalization to more effectively disrupt the adenosine barrier within the tumor microenvironment.

**Methods:**

Two monoclonal antibodies, CR201 and CR202, were generated and characterized for cross-species reactivity, inhibition of both membrane-bound and soluble CD73 enzymatic activity, and domain-specific epitope recognition. Functional assays were performed to evaluate reversal of AMP-mediated T-cell suppression, restoration of IFN-γ secretion, and induction of CD73 internalization. *In vivo* efficacy was assessed using an A375 melanoma xenograft model.

**Results:**

CR201 and CR202 specifically bound human and cynomolgus CD73 and recognized distinct structural domains, with CR202 targeting the N-terminal domain and CR201 binding the C-terminal domain. Functionally, CR202 demonstrated greater potency against membrane-bound CD73, whereas CR201 achieved complete inhibition of soluble CD73 activity. Both antibodies effectively restored T-cell proliferation and IFN-γ production and promoted internalization of surface CD73. *In vivo*, treatment with either antibody significantly suppressed tumor growth without observable toxicity.

**Conclusion:**

CR201 and CR202 are domain-specific, dual-mechanism anti-CD73 antibodies that integrate potent enzymatic blockade with receptor internalization, thereby enhancing antitumor immune responses. These findings highlight the therapeutic potential of targeting distinct functional domains of CD73 to overcome adenosine-mediated immunosuppression in cancer.

## 1 Introduction

Immune evasion is a hallmark of cancer and is largely driven by immunosuppressive mechanisms within the tumor microenvironment (TME) that impair effective antitumor immune responses (1–3). Among these, extracellular adenosine has emerged as a critical immunosuppressive metabolite that accumulates at high levels in the TME and exerts potent inhibitory effects on immune cells, particularly T lymphocytes. In addition to suppressing cytotoxic immune function, adenosine also promotes tumor cell proliferation, survival, and angiogenesis, thereby facilitating tumor progression (4).

Adenosine is primarily generated through the sequential hydrolysis of extracellular ATP by CD39 and CD73. Notably, CD73 catalyzes the terminal and irreversible step of this pathway by converting AMP into adenosine, positioning it as a central regulator of adenosine production within the TME (5–8). CD73, also known as ecto-5′-nucleotidase (5′-NT) and encoded by the NT5E gene, is a glycosylphosphatidylinositol (GPI)-anchored homodimeric ectoenzyme composed of an N-terminal metal-binding domain and a C-terminal catalytic domain connected by a flexible linker (9–12). This structural organization enables conformational transitions that are essential for enzymatic activity. In addition to its membrane-bound form, CD73 also exists as a soluble enzyme generated through GPI-anchor cleavage or proteolytic shedding, further contributing to extracellular adenosine accumulation (13–15).

Aberrant overexpression of CD73 has been reported across a wide range of malignancies, including glioblastoma, pancreatic, colorectal, and non–small cell lung cancers, where it is associated with tumor growth, metastasis, angiogenesis, and immune evasion (16–22). Mechanistically, CD73-derived adenosine signals through A2A and A2B receptors to suppress T-cell activation and effector function, thereby promoting tumor immune escape (15, 20, 23–25). Recently studies have demonstrated that genetic ablation or pharmacological inhibition of CD73 can suppress tumor growth and enhance responses to immune checkpoint blockade (26–33). These findings have led to the development of multiple CD73-targeting agents, including small-molecule inhibitors such as quemliclustat and monoclonal antibodies such as oleclumab (34–43). However, despite favorable safety profiles, these agents have shown limited efficacy as monotherapies, highlighting important limitations in current targeting strategies.

Emerging evidence suggests that the functional efficacy of CD73-targeting antibodies may be constrained by several factors, including incomplete inhibition of soluble CD73 activity, domain-dependent differences in enzymatic blockade, and the potential for reduced inhibitory potency at high antibody concentrations (the “hook effect”) (44–46). In addition, the ability of antibodies to induce CD73 internalization and reduce surface enzyme availability may represent an underexplored mechanism to enhance therapeutic efficacy. These considerations underscore the need for next-generation CD73-targeting antibodies with improved mechanistic versatility and functional potency.

Here, we report the development of two novel anti-CD73 monoclonal antibodies, CR201 and CR202, designed to address these limitations through a dual-mechanism strategy. CR202 targets the N-terminal domain of CD73, whereas CR201 binds the C-terminal domain, enabling domain-specific modulation of CD73 enzymatic function and conformational dynamics. In addition to directly inhibiting enzymatic activity, both antibodies promote receptor internalization, thereby reducing CD73 availability on the cell surface. Through comprehensive *in vitro* and *in vivo* characterization, we demonstrate that CR201 and CR202 exhibit distinct yet complementary functional profiles, supporting their potential as next-generation therapeutic candidates for targeting the adenosine axis in cancer.

## 2 Materials and methods

### 2.1 Antibody preparation

The Anti-CD73 antibody CR201 and CR202 were identified through an internal antibody discovery platform developed by China Resources Biopharmaceutical Company Limited. Sequence information is currently protected under pending patent applications (Application No. CN202211603135.8, CN202211603148.5). The heavy and light chain sequences of CR201 and CR202 were cloned into pTT5 vectors with heavy chain constant region subtype hIgG1 (N297A) and light chain constant region subtype kappa. Reference antibodies, Oleclumab and Uliledlimab, were derived from patents US 2016/0129108 A1 and US 10584169 B2, respectively, and cloned similarly into pTT5 vectors. Plasmids were co-transfected into ExpiCHO cells (A29133, Thermo Fisher Scientific, Waltham, MA, USA) using polyethylenimine (PEI)-mediated transient transfection. Nine days post-transfection, culture supernatants were harvested by centrifugation and filtered through 0.22 μm filters. The antibodies were subsequently purified using a 5 mL Protein A Resin FF prepacked column (L00680-51, GenScript, Nanjing, China) and Superdex™ 75 Increase 10/300 GL Prepacked size-exclusion chromatography columns (29148721, Cytiva, Marlborough, MA, USA).

### 2.2 Cell lines, and animals

Human breast cancer MDA-MB-231 and melanoma A375 cell lines were purchased from the American Type Culture Collection (ATCC). CD73-overexpressing CHOK1 cell lines CHOK1-huCD73, CHOK1-cynoCD73, and CHOK1-mCD73 were and provided by Shanghai ChemPartner Co., Ltd. The cells were routinely subcultured and cryopreserved in accordance with the operation manual and standard cell culture protocols. Female BALB/c nude mice (6–8 weeks old) were obtained from Shanghai Lingchang Biotechnology Co., Ltd. Mice were housed in SPF-grade facilities within individually ventilated cages. All procedures were approved by the local Institutional Animal Care and Use Committee (IACUC) and conducted in accordance with institutional guidelines. The animal study was approved by the Institutional Animal Care and Use Committee (IACUC) of Shanghai Rui Zhi Chemical Research Co., Ltd. (B16-4-20210901-0090-20240901).

### 2.3 Cell binding assay

CHOK1-huCD73, CHOK1-cynoCD73, and CHOK1-mCD73 cells were harvested and washed twice with FACS buffer (PBS + 2% FBS). For each assay, 5 × 10^5^ cells/well were aliquoted into 96-well plates and incubated with serially diluted anti-CD73 antibodies (starting concentration: 100 nM, 5-fold serial dilutions, 7 concentrations) for 1 h at 4 °C. Cells were washed twice with FACS buffer and incubated with Goat Anti-Human IgG Fc-Alexa Fluor 647 antibody (109-606-170, Jackson ImmunoResearch West Grove, PA, USA) staining for 1 h at 4 °C. After washing, fluorescence intensity was measured using a BD Aria III flow cytometer (BD Biosciences, San Jose, CA, USA). EC_50_ values were calculated using four-parameter nonlinear regression.

### 2.4 CD73 enzymatic activity inhibition assay

Recombinant CD73 protein or cell suspensions (CHOK1-huCD73 or MDA-MB-231) were resuspended in TM buffer (25 mM Tris, 5 mM MgCl_2_, pH 7.5) and plated into 96-well plates at a density of 5 × 10^4^ cells per well or 5 nM recombinant CD73 per well. Serially diluted anti-CD73 antibodies were added to the wells and incubated at 37 °C for 30 min (starting concentration: 100 nM, 5-fold serial dilutions, 7 concentrations; n = 2). 500 nM of AMP was then added to the plates followed by 37°C incubation for 30 min. Supernatants were transferred to new 96-well plates, mixed with ATP solution diluted in TM buffer, and supplemented with CellTiter-Glo Reagent (G7573, Promega, Madison, WI, USA). Luminescence was measured after 10 min at room temperature using a microplate reader to quantify CD73 enzymatic activity.

### 2.5 AMP-mediated T cell suppression release assay

CD4^+^ T cells were isolated from human peripheral blood mononuclear cells (PBMCs) using the EasySep™ Human CD4^+^ T Cell Isolation Kit (17952, Stemcell, Vancouver, BC, Canada). Isolated cells were labeled with 2.5 μM CFSE (C34554, Thermo Fisher Scientific) by incubating in RPMI-1640 medium with 10% FBS at 37 °C for 10 minutes. The staining reaction was quenched by adding an equal volume of RPMI-1640 containing 40% FBS and incubating for 5 minutes at room temperature. Cells were then washed twice and resuspended at 1 × 10^6^ cells/mL. T cells were stimulated with anti-CD2/CD3/CD28 activator beads (11132D, Thermo Fisher Scientific) at a bead-to-cell ratio of 1:1 and cultured in RPMI-1640 supplemented with 10% FBS. Serial dilutions of anti-CD73 antibodies (CR201, CR202) (Antibody concentration: 50nM, 16.67nM, 3.33nM, 0.67nM, 0.13nM, 0.03nM, 0.01nM, 0; n=2) and 500nM AMP were added to the wells, and cells were incubated at 37 °C in 5% CO_2_ for 96 hours. T cell proliferation was analyzed by measuring CFSE dilution using a BD FACSCanto II flow cytometer. Data were processed using FlowJo software (FlowJo v10.8.1), and proliferation indices were calculated relative to the isotype control.

### 2.6 Antibody-mediated target internalization assay

CHOK1-huCD73 cells were plated at 1 × 10^5^ cells per well in 96-well plates and incubated with 10nM anti-CD73 antibodies at 4 °C for 30 minutes. Unbound antibodies were removed by washing twice with FACS buffer. Cells were resuspended and subjected to internalization at 4°C or 37°C for 1, 2, and 4 hours. At each time-temperature point, cells were fixed with 2% paraformaldehyde fixative on ice for 15 min. After supernatant removal, cells were incubated with Goat Anti-Human IgG Fc-Alexa Fluor 647 antibody at 4°C for 1 hour. Finally, cells were resuspended in PBS and analyzed by flow cytometry. After washing, residual surface CD73 was quantified by flow cytometry.

### 2.7 Mixed lymphocyte reaction

Fresh PBMCs (Junx Bio, Shanghai, China) were resuspended in serum-free RPMI-1640 medium at a density of 1 × 10^6^ cells/mL and incubated at 37 °C for 2–3 hours to allow monocyte adherence. Non-adherent cells were removed, and adherent cells were cultured in RPMI-1640 supplemented with 250 U/mL IL-4, 500 U/mL GM-CSF, and 10% FBS to induce differentiation into immature dendritic cells (DCs). Half-medium changes were performed every 48 hours. On day 6, 1 μg/mL lipopolysaccharide (LPS) was added to induce DC maturation. Mature DCs (stimulators) were treated with 10 μg/mL mitomycin C at 37 °C for 45 minutes to inhibit proliferation, followed by washing prior to co-culture. CD4^+^ T cells (responders) were isolated from human PBMCs. DCs and CD4^+^ T cells were co-cultured in 96-well plates at a ratio of 1:20 (DC:T cells) in RPMI-1640 containing 10% FBS, in the presence of anti-PD-1 antibody, AMP, and anti-CD73 antibodies (CR201 or CR202) at the indicated concentrations. After 120 hours of incubation at 37 °C with 5% CO_2_, culture supernatants were collected, and IFN-γ levels were quantified using the IFN-γ HTRF assay kit (62HIFNGPEG, Cisbio, Codolet, France) according to the manufacturer’s instructions.

### 2.8 Epitope binning

Epitope binning was performed using a flow cytometry–based competitive binding assay. CHOK1-huCD73 cells stably expressing human CD73 were harvested and resuspended in FACS buffer at a density of 1 × 10^5^ cells per well in 96-well plates. Cells were first incubated with unlabeled (blocking) antibodies at a concentration of 40 μg/mL at 4 °C for 30 minutes to allow competitive binding. Without washing, fluorescence-conjugated detection antibodies were added at saturating concentrations and incubated for an additional 45 minutes at 4 °C. Following incubation, cells were washed three times with FACS buffer and resuspended in PBS for analysis.

### 2.9 Affinity analysis

Binding affinities were measured by surface plasmon resonance (SPR) using a Biacore T200 instrument (Cytiva, Uppsala, Sweden). A CM5 sensor chip was immobilized with anti-human IgG by amine coupling. Antibodies (CR201 and CR202) were captured onto the chip surface. Serial dilutions of recombinant human CD73-His were injected at a flow rate of 30 μL/min. Association and dissociation were monitored for 180 s and 420 s, respectively. The surface was regenerated with 10 mM glycine-HCl (pH 2.0). Kinetic parameters were determined using a 1:1 Langmuir binding model with Biacore T200 Evaluation Software.

### 2.10 *In vivo* antitumor activity

Subcutaneous xenograft models were established in 6–8-week-old female BALB/c nude mice by injecting 4 × 10^6^ A375 cells in 200 μL DMEM into the right dorsal flank. Tumor volume was calculated as (length×width^2^)/2. When the mean tumor volume reached approximately 100 mm³ (three weeks after implantation), mice were randomized into three treatment groups, with 8 mice per group (n = 8/group). Tumor volumes and body weights were measured longitudinally throughout the study, and all timepoint analyses were based on 8 animals per group. Antibodies were administered intravenously at 10 mg/kg, three times per week for a total of 9 doses. Control groups received an equivalent dose of isotype-matched antibody. Tumor volumes and body weights were monitored throughout the study. Tumor growth inhibition (TGI) was calculated as:

(1) $$\text{TGI}(\%)=[1-({\text{TV}}_{\text{treatment-Dn}}{\text{‐TV}}_{\text{treatment}-\text{D}0}/({\text{TV}}_{\text{control‐Dn}}{\text{‐TV}}_{\text{control‐D}0})]\times 100$$

where TV_treatment_ and TV_control_​ represent the mean tumor volumes of the treatment and control groups, respectively.

### 2.11 Statistical analysis

Statistical significance was determined using GraphPad Prism software, with two-way ANOVA determining significance versus controls. Data are presented as the mean ± standard deviation(SD), and differences between groups were considered statistically significant when p < 0.05 (*p < 0.05; **p < 0.01, ***p < 0.001, ****p < 0.0001).

## 3 Results

### 3.1 CR201 and CR202 specifically bind human and cynomolgus monkey CD73

**Figure 1:**
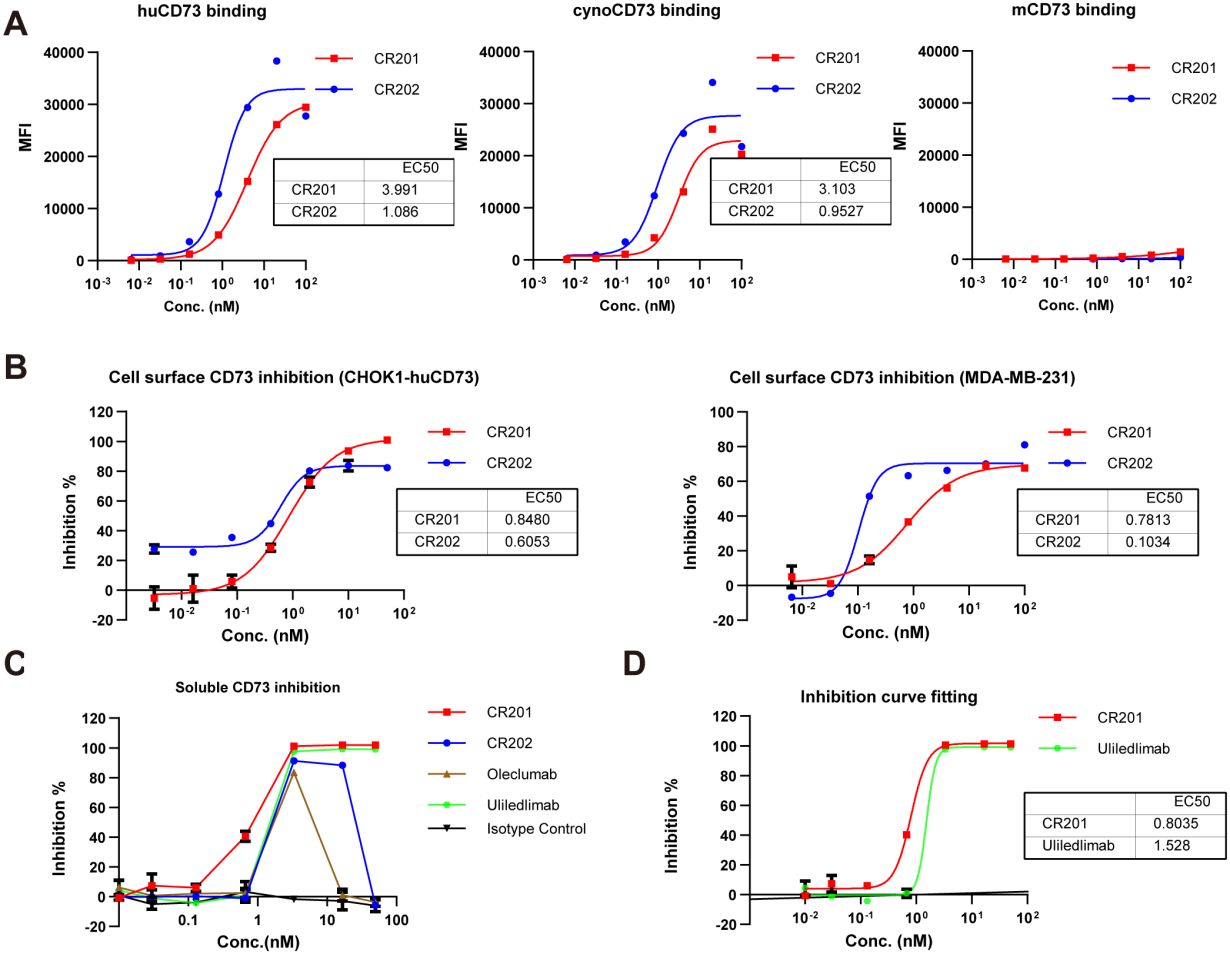
CR201 and CR202 bind CD73 and inhibit enzymatic activity. **(A)** Dose-dependent binding of CR201 and CR202 to CHOK1 cells expressing human (huCD73), cynomolgus (cynoCD73), or mouse (mCD73) CD73, measured by flow cytometry. Data are shown as mean fluorescence intensity (MFI, n=2). Antibody starting concentration: 100 nM, 5-fold serial dilutions, 7 concentrations; IgG1 isotype served as negative control. **(B)** Inhibition of cell surface CD73 enzymatic activity in CHOK1-huCD73 and MDA-MB-231 cells by CR201 and CR202. Enzymatic activity is expressed as percent of untreated control (n=2). Starting concentrations: 50 nM (CHO-K1), 100 nM (MDA-MB-231); 5-fold serial dilutions, 7 concentrations. **(C)** Inhibition of soluble recombinant CD73 by CR201, CR202, oleclumab, and uliledlimab. **(D)** Corresponding four-parameter fit curves comparing CR201 and uliledlimab. Enzymatic activity is expressed relative to IgG1 control (n=2). Antibody concentrations: 50, 16.67, 3.33, 0.67, 0.13, 0.03, 0.01, 0 nM.

Flow cytometry analysis of CHOK1 cells expressing human, cynomolgus, or mouse CD73 revealed distinct cross-species reactivity. CR201 selectively bound human and cynomolgus CD73 (EC_50_: 3.991 nM and 3.103 nM) but not mouse CD73. CR202 showed higher potency toward human and cynomolgus CD73 (EC_50_: 1.086 nM and 0.953 nM) while failing to recognize mouse CD73 (**Figure 1A**).

### 3.2 CR201 and CR202 potently inhibit enzymatic activity of cell surface CD73

CD73 catalyzes the conversion of AMP to adenosine and is highly expressed on tumor and immunosuppressive cells. We first assessed antibody-mediated inhibition using CHOK1-huCD73 cells. Dose-response analysis showed that CR201 achieved complete inhibition at higher concentrations, whereas CR202 displayed greater potency, suppressing approximately 30% of enzymatic activity even at low concentrations (**Figure 1B**). These results were confirmed in MDA-MB-231 human breast cancer cells, which endogenously express CD73. Both antibodies effectively inhibited tumor cell surface CD73, with CR202 demonstrating significantly stronger suppression than CR201 across the tested concentrations (**Figure 1B**).

### 3.3 CR201 and CR202 potently inhibit enzymatic activity of soluble CD73

Soluble CD73 contributes to adenosine-mediated immunosuppression in the tumor microenvironment. We evaluated CR201 and CR202 against recombinant soluble CD73, using two clinical-stage anti-CD73 antibodies, oleclumab and uliledlimab, for comparison. CR201 and CR202 exhibited distinct functional profiles. CR201, similar to uliledlimab, fully suppressed CD73 enzymatic activity even at high concentrations (50 nM). In contrast, CR202, resembling oleclumab, showed a concentration-dependent decrease in inhibition at 50 nM, indicative of a hook effect (**Figure 1C**). Quantitative analysis confirmed that CR201 was more potent (IC_50_ = 0.8 nM) than uliledlimab (IC_50_ = 1.5 nM) (**Figure 1D**). At 17 nM, CR202 maintained substantial enzyme inhibition, whereas oleclumab exhibited near-complete loss of activity (**Figure 1C**).

### 3.4 CR201 and CR202 recognize distinct epitopes on CD73 antigen

**Figure 2:**
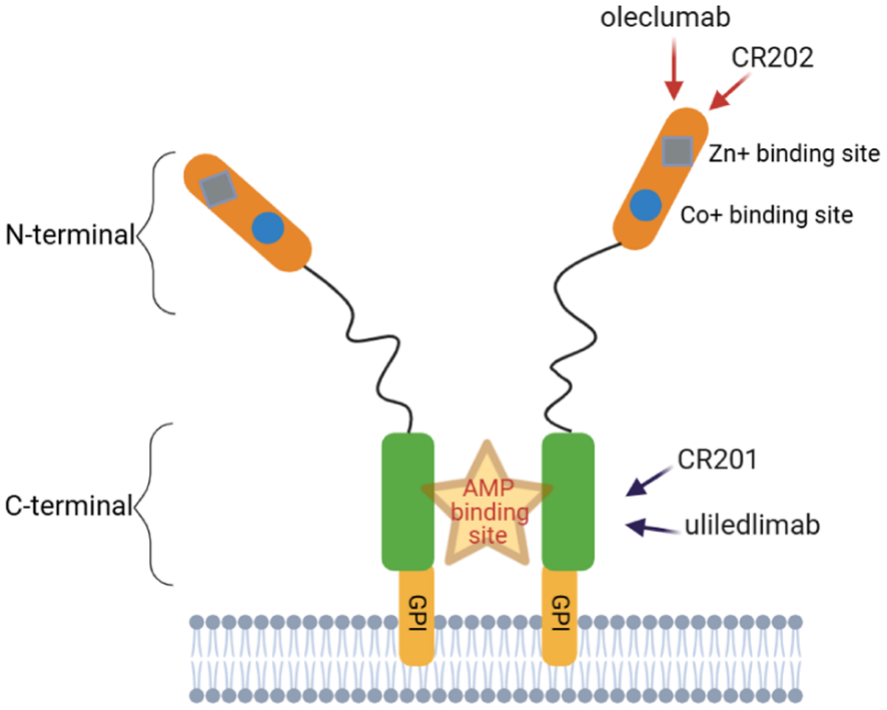
Epitope mapping of anti-CD73 antibody.

To investigate the mechanistic basis for the differential inhibition of soluble CD73, we performed epitope competition assays using clinical-stage antibodies oleclumab and uliledlimab as references. CR201 competed with uliledlimab, whereas CR202 competed with oleclumab (**Table 1**). These results suggest that CR201 targets the C-terminal domain of CD73, similar to uliledlimab, while CR202 binds the N-terminal domain, akin to oleclumab (**Figure 2**). This epitope mapping is consistent with the observed differences in soluble enzyme inhibition between the two antibodies.

**Table 1:** Epitope competition of CR201 and CR202 with reference antibodies.

	Primary abs:	Primary abs:	Primary abs:	Primary abs
Conjugated abs	CR201	CR202	Oleclumab	Uliledlimab
CR201-Alexa488	99%	51%	12%	37%
CR202-Alexa488	13%	100%	99%	10%
oleclumab- Alexa 488	-3%	100%	100%	-3%
uliledlimab- Alexa 488	82%	52%	10%	98%

### 3.5 CR202 potently reverses AMP-induced suppression of T-cell proliferation

**Figure 3:**
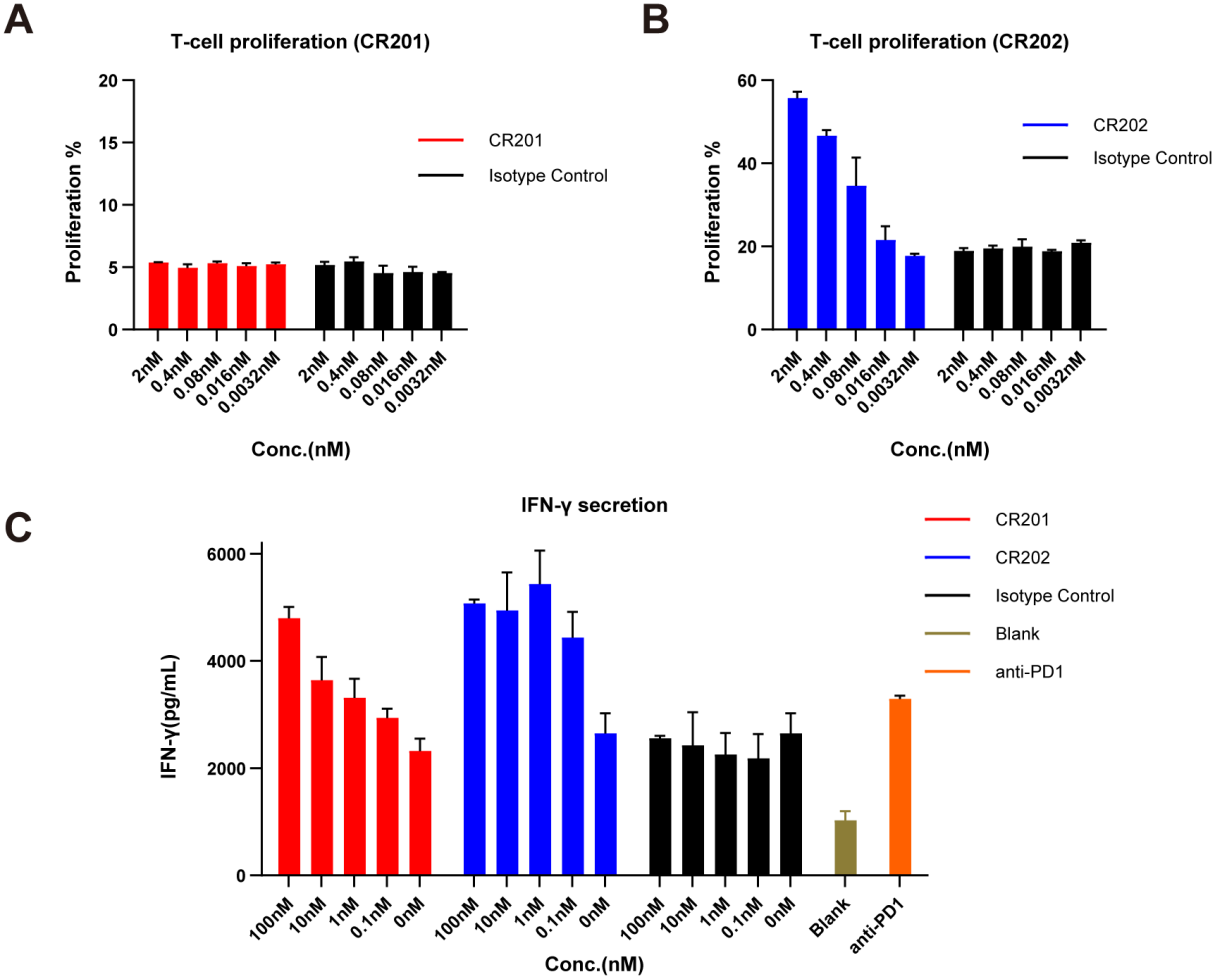
CR201 and CR202 restore T-cell function under AMP-mediated immunosuppression. **(A, B)** Reversal of AMP-induced suppression of T-cell proliferation by CR201 **(A)** and CR202 **(B)**. Proliferation is expressed as percent of untreated T cells (n=2). Antibody starting concentration: 2 nM, 5-fold serial dilutions, 5 concentrations; AMP: 500 nM; IgG1 isotype included as negative control. **(C)** Restoration of IFN-γ secretion in CD4^+^ T cells under AMP-mediated suppression. Data are presented as percent of control secretion (n=2). Experimental conditions as in **(A)**.

Adenosine produced by CD73-catalyzed AMP hydrolysis suppresses T-cell proliferation. To assess the capacity of CR201 and CR202 to counteract this effect, we evaluated their ability to reverse AMP-induced T-cell suppression. Representative flow cytometry gating strategies are shown in **Supplementary Figure 1**. CR202 significantly restored T-cell proliferation in a dose-dependent manner (**Figure 3A**), whereas CR201 exhibited minimal activity, comparable to the isotype control (**Figure 3B**).

### 3.6 CR201 and CR202 effectively reverse AMP-induced suppression of IFN-γ secretion in CD4^+^ T cells

CD73-mediated AMP-to-adenosine conversion suppresses IFN-γ secretion in CD4^+^ T cells, contributing to immunosuppression in the tumor microenvironment. Mature dendritic cells (DCs) expressing PD-L1 inhibit PD-1^+^ CD4^+^ T-cell activity, an effect partially reversed by anti-PD-1 antibodies. Elevated AMP levels, however, counteract this restoration by suppressing IFN-γ production. Both CR201 and CR202 effectively blocked AMP-induced immunosuppression, restoring IFN-γ secretion in a dose-dependent manner (**Figure 3C**).

### 3.7 CR201 and CR202 effectively induce cell surface CD73 internalization

**Figure 4:**
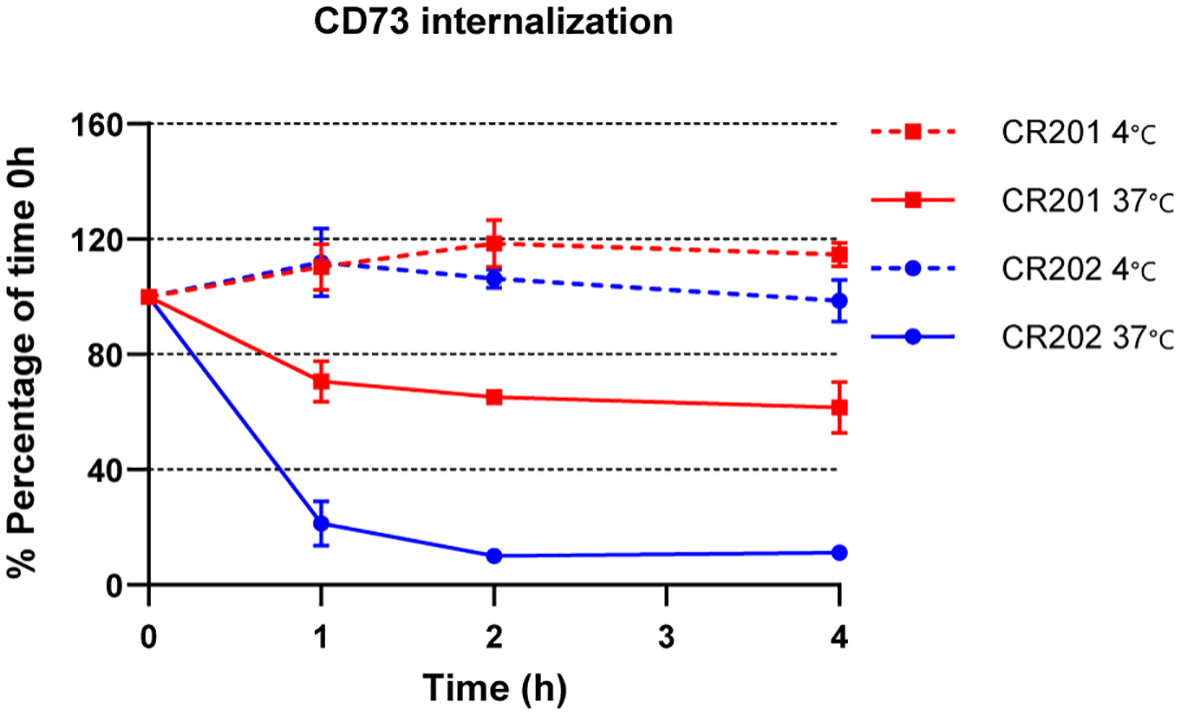
CR201 and CR202 induce internalization of cell surface CD73. Time-dependent reduction of surface CD73 in CHOK1-huCD73 cells, measured by flow cytometry over 0, 1, 2, and 4 hours at 37 °C. Data are expressed as percent reduction of surface CD73 relative to 0 h (n=2). CR202 induced greater internalization than CR201.

As a membrane-anchored ectoenzyme, CD73 catalyzes AMP hydrolysis to adenosine. Antibody-mediated internalization reduces surface CD73, thereby limiting adenosine production. To evaluate this effect, we measured residual membrane-bound CD73 via flow cytometry following temperature-controlled incubation. Both CR201 and CR202 promoted CD73 internalization. After 4 hours at 37 °C, CR202 induced 88.8% internalization, whereas CR201 mediated a 38.4% reduction in surface CD73 (**Figure 4**). These results highlight their dual mechanism of action: enzymatic inhibition combined with receptor internalization, enhancing biological efficacy.

### 3.8 CR201 and CR202 exhibit high affinity for CD73

**Figure 5:**
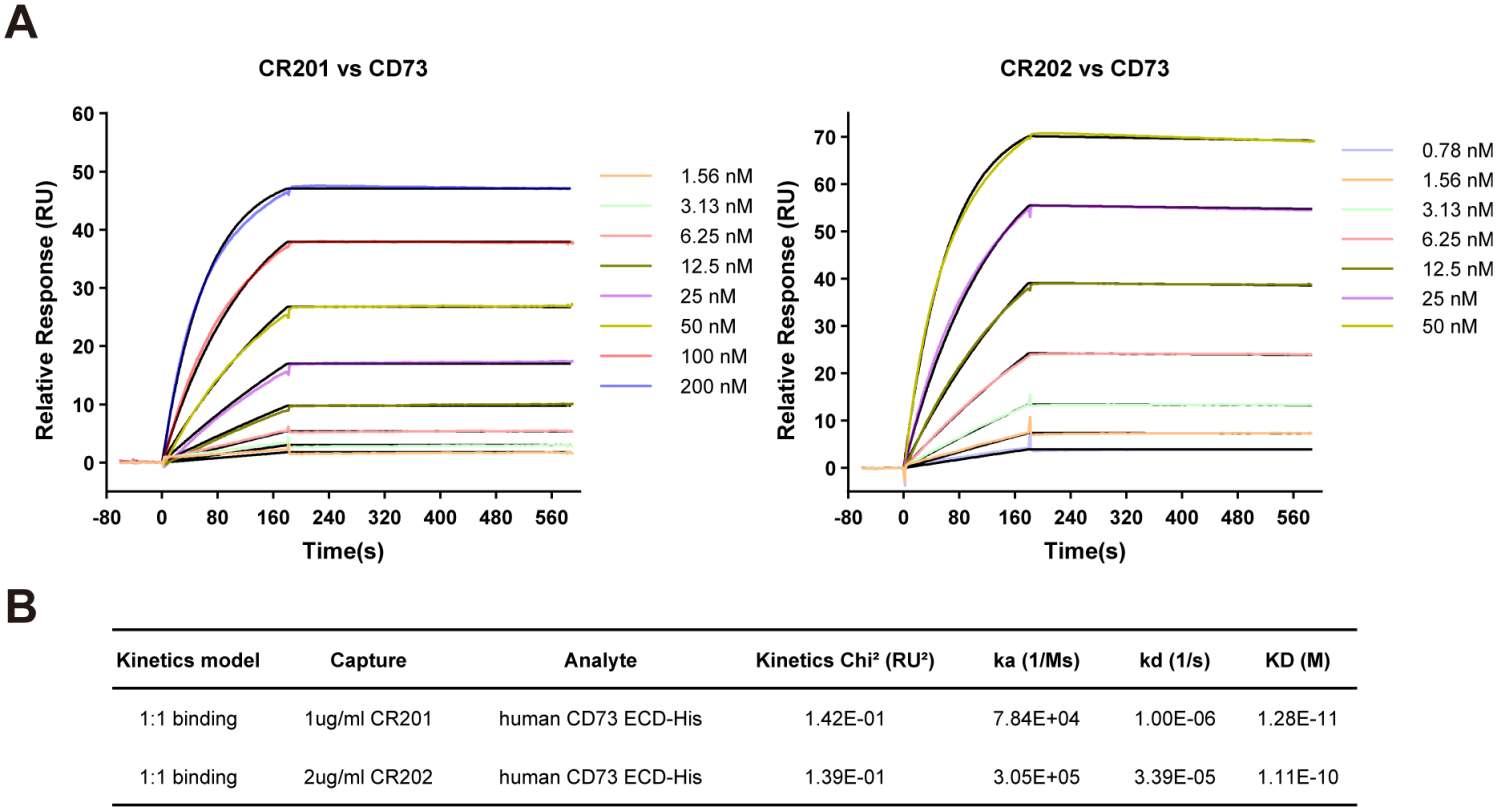
Binding affinity of CR201 and CR202 to human CD73. **(A)** Representative SPR sensorgrams of CR201 (left) and CR202 (right) binding to recombinant human CD73. **(B)** Kinetic parameters (KD, kon, koff) derived from SPR measurements, quantifying antibody-target interaction strength and binding dynamics (n=2).

The binding affinities of CR201 and CR202 were assessed using SPR. CR201 demonstrated high affinity for CD73, with a KD of 1.28 × 10^-^¹¹ M, while CR202 also exhibited strong binding, with a KD of 1.11 × 10^-^¹^0^ M (**Figure 5**, **Table 2**).

**Table 2:** Antitumor efficacy of test articles in the A375 xenograft model (tumor volume).

Group	n	Body weight (g) ^a^		Tumor volume (mm³) ^a^		TGI_TV_ (%)	*p ^b^*
		Day 0	Day 22	Day 0	Day 22		
Isotype Control	8	21.17 ± 0.35	22.08 ± 0.53	101.79 ± 18.97	2405.38 ± 367.50	‐-	‐-
CR201	8	20.78 ± 0.32	21.71 ± 0.63	98.32 ± 15.15	1488.23 ± 286.22	40%	0.0001
CR202	8	20.51 ± 0.25	22.02 ± 0.61	97.06 ± 19.18	1471.58 ± 145.58	40%	<0.0001

^a^Data are expressed as mean ± SEM. *p*-values were calculated based on tumor growth curves using two-way ANOVA followed by Tukey’s multiple comparisons test. *P < 0.05, **P < 0.01, ***P < 0.001. TGI_TV_ (%), Tumor growth inhibition based on tumor volume.

### 3.9 Anti-tumor efficacy in A375 melanoma xenografts in BALB/c nude mice

**Figure 6:**
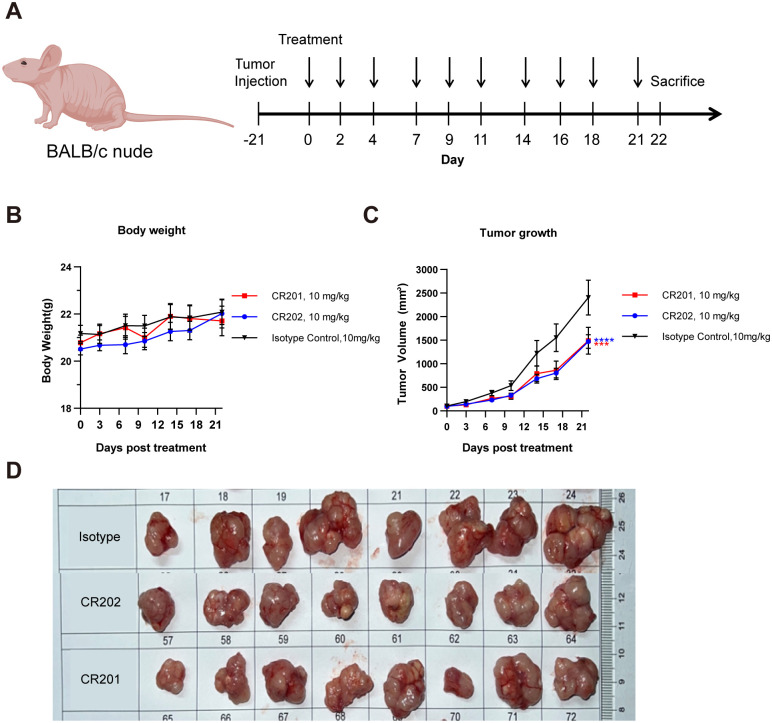
*In vivo* antitumor efficacy of CR201 and CR202 in A375 xenograft model. **(A)** Experimental timeline for antibody treatment in A375 xenograft mice. **(B)** Body weight changes during treatment. Data are presented as mean ± SD from 8 mice per group at each timepoint. **(C)** Tumor growth curves over the treatment period. Data are presented as mean ± SD from 8 mice per group at each timepoint. **(D)** Final tumor images at study endpoint. Tumor growth data were analyzed using two-way ANOVA followed by Tukey’s multiple comparisons test.

The *in vivo* antitumor efficacy of CR201 and CR202 was evaluated in subcutaneous xenografts of CD73-high A375 melanoma cells in immunodeficient BALB/c nude mice (**Figure 6A**). Both antibodies significantly inhibited tumor growth by Day 22 post-treatment compared to controls (p < 0.0001) (**Table 2**). No significant differences in body weight were observed between treated and control groups, indicating favorable tolerability for both CR201 and CR202 (**Figure 6B**). Tumor growth kinetics demonstrated potent and sustained antitumor activity, with both antibodies effectively restraining A375 tumor progression relative to isotype controls (**Figures 6C, D**).

## 4 Discussion

Adenosine is a key immunosuppressive molecule in the tumor microenvironment (TME), inhibiting tumor-infiltrating immune cells through cell surface adenosine receptors. Despite their broad expression, the distinct biological roles of these receptors remain incompletely characterized (37). Within the ATP-to-adenosine metabolic pathway, the conversion of AMP to adenosine by CD73 represents the sole irreversible step. AMP accumulation in the TME arises via multiple enzymatic pathways beyond CD39-mediated ATP hydrolysis, underscoring the mechanistic advantage of targeting CD73 to disrupt adenosine production at its terminal node.

Previous studies have shown that CD73-blocking antibodies inhibit both membrane-bound and soluble CD73. Notably, soluble CD73 inhibition falls into two categories: one class exhibits reduced efficacy at high antibody concentrations (e.g., oleclumab), while the other maintains consistent inhibition (e.g., uliledlimab). This difference reflects the butterfly-like configuration of the CD73 dimer (**Figure 4**). AMP binds at the interface of the two subunits, and catalysis requires folding of the N-terminal domain toward the C-terminal domain. Antibodies targeting the N-terminal domain inhibit catalysis by locking the two “wings” of a single dimer. At high concentrations, however, these antibodies may crosslink separate dimers or bind only one monomer, resulting in loss of catalytic inhibition—the so-called “hook effect”.

In this study, we characterized two novel CD73 antibodies, CR201 and CR202, using comprehensive *in vitro* and *in vivo* assays. CR202 (N-terminal binder) and CR201 (C-terminal binder) selectively bound human and cynomolgus CD73, without cross-reactivity to murine CD73. In CHO-K1 cells overexpressing CD73, CR201 completely inhibited enzymatic activity, while CR202 achieved ~30% inhibition at picomolar concentrations. In naturally CD73-high MDA-MB-231 cells, CR202 exhibited superior, dose-dependent inhibition compared to CR201. Interestingly, despite its potent inhibition of soluble CD73 activity, CR201 showed limited reversal of AMP-mediated T-cell proliferation suppression compared with CR202. This difference may reflect distinct functional requirements among immune assays and suggests that membrane CD73 regulation and receptor internalization could play a more prominent role in certain cellular contexts.

Recent studies have suggested that antibody-mediated CD73 internalization may represent an important complementary mechanism to direct enzymatic inhibition. In addition to blocking CD73 catalytic activity, reduction of membrane-associated CD73 through receptor internalization may provide more sustained suppression of extracellular adenosine production within the tumor microenvironment. Accordingly, increasing attention has been given to multifunctional CD73-targeting strategies that combine enzymatic inhibition with receptor internalization or depletion. Several recent studies, including bispecific and Fc-conjugated CD73-targeting antibodies, have reported enhanced antitumor activity associated with these dual mechanisms (47–49). In the current study, both CR201 and CR202 effectively induced CD73 internalization while simultaneously inhibiting enzymatic activity, suggesting that these antibodies share features consistent with emerging multifunctional anti-CD73 therapeutic strategies.

For soluble CD73, CR202 displayed a concentration-dependent reduction in inhibition at high antibody levels, consistent with the hook effect, whereas CR201 maintained full inhibition without attenuation, aligning with previous reports (37, 50). Functional assays demonstrated that CR202 more effectively reversed AMP-mediated suppression of T-cell proliferation and AMP-induced inhibition of IFN-γ secretion in CD4^+^ T cells. Interestingly, *in vivo*, CR201 exhibited tumor growth inhibition comparable to CR202, despite its lower *in vitro* immunomodulatory potency. However, interpretation of these findings should be made with caution, as the BALB/c nude mouse model lacks a fully functional adaptive immune system and therefore may not fully recapitulate T cell-mediated antitumor immunity. Under these conditions, the observed antitumor effects may reflect broader modulation of the CD73-adenosine axis, including inhibition of membrane-bound and soluble CD73 activity, rather than differences in adaptive immune activation alone. Although the physiological roles of soluble CD73 remain incompletely defined, evidence suggests involvement in critical processes such as vascular integrity (27, 51). Further studies using immunocompetent or humanized *in vivo* models will be necessary to better define the relative contribution of enzymatic inhibition, receptor internalization, and immune modulation to the antitumor effects of CR201 and CR202.

In summary, this study presents two novel human CD73 antibodies, CR202 (N-terminal binder) and CR201 (C-terminal binder), which act via a dual mechanism: enzymatic blockade, directly inhibiting CD73 catalytic activity to reduce adenosine generation, and receptor internalization, mediating surface CD73 clearance to further suppress enzymatic function. These findings support the potential of CR201 and CR202 to modulate the immunosuppressive tumor microenvironment.

Several limitations of the current study should be acknowledged. First, the *in vivo* antitumor activity of CR201 and CR202 was evaluated using immunodeficient nude mouse xenograft models, which do not fully recapitulate the complexity of human antitumor immune responses. Future studies using humanized or transgenic CD73-expressing models will be important to further investigate the immunomodulatory effects of these antibodies within the tumor microenvironment. In addition, although both CR201 and CR202 exhibited dual functional properties involving enzymatic inhibition and CD73 internalization, the relative contribution of these mechanisms to antitumor efficacy was not specifically dissected in the present study. Finally, tolerability assessment in this work was primarily based on body weight monitoring, and more comprehensive pharmacokinetic, histopathological, and toxicological evaluations will be required to further characterize the safety and pharmacological profiles of CR201 and CR202 in future preclinical studies.

## Data Availability

The original contributions presented in the study are included in the article/**Supplementary Material**. Further inquiries can be directed to the corresponding authors.
