# Supplementary material for: Dual-mechanism anti-CD73 antibodies CR201 and CR202 targeting distinct domains for cancer immunotherapy
Source: Front Immunol. 2026 Jun 10;17:1861537. doi: 10.3389/fimmu.2026.1861537 (PMC13291015; doi:10.3389/fimmu.2026.1861537)
Supplement: Supplementary file 1 [file Image1.pdf]

Supplementary Figure S1

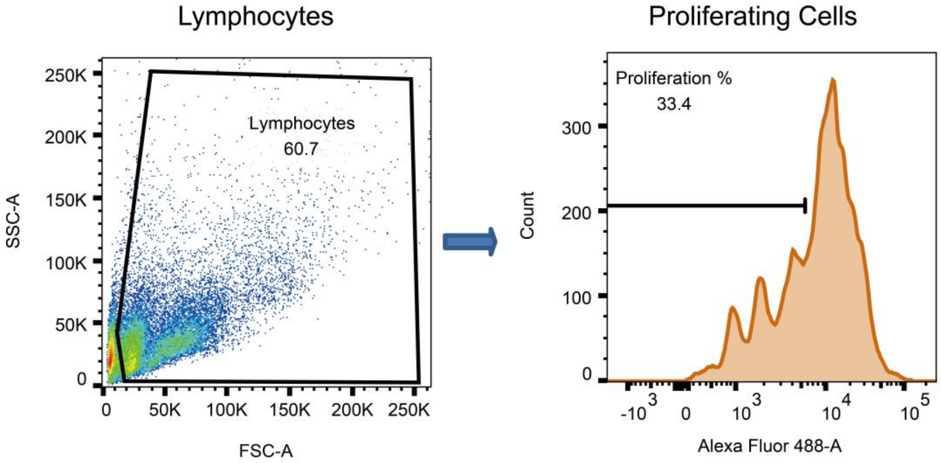

Figure S1. Representative gating strategy for the CFSE-based T-cell proliferation assay. Representative gating strategy for the CFSE-based T-cell proliferation assay. Lymphocytes were gated based on FSC/SSC characteristics. Proliferating T cells were identified as the CFSE-low population..
